# Spontaneous Post-Transplant Disorders in NOD.Cg- *Prkdc^scid^ Il2rg^tm1Sug^*/JicTac (NOG) Mice Engrafted with Patient-Derived Metastatic Melanomas

**DOI:** 10.1371/journal.pone.0124974

**Published:** 2015-05-21

**Authors:** Enrico Radaelli, Els Hermans, Lorna Omodho, Annick Francis, Sara Vander Borght, Jean-Christophe Marine, Joost van den Oord, Frédéric Amant

**Affiliations:** 1 VIB11 Center for the Biology of Disease, KU Leuven Center for Human Genetics, Leuven, Belgium; 2 InfraMouse, KU Leuven-VIB, Leuven, Belgium; 3 Gynaecological Oncology, UZ Leuven—Department of Oncology, KU Leuven, Leuven, Belgium; 4 Department of Pathology, Laboratory of Morphology and Molecular Pathology, University Hospitals of Leuven, Leuven, Belgium; 5 Laboratory for Molecular Cancer Biology, VIB11 Center for the Biology of Disease, KU Leuven Center for Human Genetics, Leuven, Belgium; 6 Translational Cell and Tissue Research, Department of Imaging and Pathology, KU Leuven, Leuven, Belgium; Rutgers University, UNITED STATES

## Abstract

Patient-derived tumor xenograft (PDTX) approach is nowadays considered a reliable preclinical model to study *in vivo* cancer biology and therapeutic response. NOD scid and *Il2rg*-deficient mice represent the “gold standard” host for the generation of PDTXs. Compared to other immunocompromised murine lines, these mice offers several advantages including higher engraftment rate, longer lifespan and improved morphological and molecular preservation of patient-derived neoplasms. Here we describe a spectrum of previously uncharacterized post-transplant disorders affecting 14/116 (12%) NOD.Cg- *Prkdc^scid^ Il2rg^tm1Sug^*/JicTac (NOG) mice subcutaneously engrafted with patient-derived metastatic melanomas. Affected mice exhibited extensive scaling/crusting dermatitis (13/14) associated with emaciation (13/14) and poor/unsuccessful tumor engraftment (14/14). In this context, the following pathological conditions have been recognized and characterized in details: (i) immunoinflammatory disorders with features of graft versus host disease (14/14); (ii) reactive lymphoid infiltrates effacing xenografted tumors (8/14); (iii) post-transplant B cell lymphomas associated with Epstein-Barr virus reactivation (2/14). We demonstrate that all these entities are driven by co-transplanted human immune cells populating patient-derived tumor samples. Since the exploding interest in the utilization of NOD scid and *Il2rg*-deficient mice for the establishment of PDTX platforms, it is of uppermost importance to raise the awareness of the limitations associated with this model. The disorders here described adversely impact tumor engraftment rate and animal lifespan, potentially representing a major confounding factor in the context of efficacy and personalized therapy studies. The occurrence of these conditions in the NOG model reflects the ability of this mouse line to promote efficient engraftment of human immune cells. Co-transplanted human lymphoid cells have indeed the potential to colonize the recipient mouse initiating the post-transplant conditions here reported. On the other hand, the evidence of an immune response of human origin against the xenotransplanted melanoma opens intriguing perspectives for the establishment of suitable preclinical models of anti-melanoma immunotherapy.

## Introduction

Patient-derived tumor xenograft (PDTX) approach is nowadays considered one of the most accurate preclinical tools to study *in vivo* biology and therapeutic response of individual human cancers [1,2]. A variety of PDTX models have been successfully established for preclinical/clinical drug testing and biomarker identification in diverse human neoplasms including ovarian, pancreatic, breast, skin and prostate cancers [2–7]. In this context, PDTX approach has been shown to be biologically stable and accurately reflect the original patient tumor with regards to expression profile, mutational spectrum and molecular signaling [4,5,8].

NOD scid mice with *Il2rg* deficiency [i. e. NOD.Cg-*Prkdc* ^*scid*^ *Il2rg* ^*tm1Wjl*^/SzJ (NSG mice from The Jackson Laboratory) and NOD.Cg- *Prkdc* ^*scid*^ *Il2rg* ^*tm1Sug*^/JicTac (NOG mice from Taconic)] combine several genetic defects that primarily affect the immune system leading to a severe impairment of both innate and adaptive immune response. NOD background is characterized by a loss of function mutation in the C5 hemolytic complement gene and by a unique MHC haplotype, which leads to impaired natural killer cell function and defects in antigen-presenting activity. Homozygosity for the *Prkdc* ^*scid*^ mutation results in defective somatic recombination at T cell receptor and immunoglobulin chains loci with consequent defective development and maturation of T and B cell clones. The targeted mutation in the γ chain of the IL-2 receptor leads to deficiencies in cytokine signaling and failure of clonal lymphocyte expansion [9–14].

Thanks to their profound immunodeficiency status, NSG and NOG mice represent the “gold standard” host for xenotransplantation experiments including patient-derived tumor biopsies. When compared to other immunocompromised murine lines, NSG and NOG mice exhibit higher PDTX engraftment rate and improved preservation of original patient tumor in terms of morphological features, tumor microenvironment and cellular heterogeneity [15–18]. Additional advantages of NSG and NOG mice over the other immunodeficient strains include lower predisposition for the development of spontaneous tumors, longer lifespan and no T and/or B cell “leakiness” documented so far [10,12,14].

Recent studies demonstrated the relevance of NOD scid mice with deficient *Il2rg* for the definition of phenotypic heterogeneity, tumorigenicity and metastatic potential of patient-derived melanomas *in vivo* [19–21]. Based on these promising results, the PDTX platform at University Hospitals Leuven established a NOG model-based pipeline with the aim to provide interested research groups with an invaluable preclinical tool to dissect the molecular landscapes that drive melanoma development and progression and test novel targeted therapeutic strategies on a large scale [2].

In this work, we characterize at clinical, pathological and molecular level the development of a spectrum of previously uncharacterized post-transplant disorders affecting NOG mice engrafted with patient-derived metastatic melanomas. All these pathological conditions are driven by co-transplanted human immune cells populating patient-derived tumor samples and represent important limitations in the context of PDTX models as they adversely impact on tumor engraftment rate and animal lifespan.

## Materials and Methods

### Collection, examination and xenotransplantation of human tumor biopsies

All the original melanoma biopsies included in the PDTX platform were received freshly from the operation theatre and promptly processed as follow. One representative part was fixed in 10% neutral buffered formalin, and used for routine histopathological diagnosis. A second portion immediately adjacent to the one selected for histopathology was used for xenotransplantation. To ensure that the tumor was adequately represented in this latter specimen, histopathology was also performed on thin tissue slices obtained from the sample destined for xenotransplantation (S1 Table). The remainder of the biopsy was snap frozen in liquid nitrogen-cooled isopentane and stored at -80°C. The portion of the biopsy selected for xenotransplantation was transported in RPMI 1640 medium supplemented with penicillin (100 U/ml), streptomycin (100 μg/ml), fungizone (1 μg/ml) and gentamicin (50 μg/ml) all from Life Technologies. The sample was then briefly rinsed PBS (also supplemented with penicillin, streptomycin and fungizone at the same concentrations) and minced into minute tissue fragments which were eventually inoculated subcutaneously in the interscapular fat pad of 6-week-old NOG females. Procedures for collection, preparation and transplantation of tumor biopsies were performed under sterile conditions.

Regarding the 6 metastatic melanomas (i.e. 4 cases of nodal metastasis, 1 case of in transit metastasis and 1 case of hepatic metastasis) considered in this study, serial sections obtained from formalin-fixed and paraffin-embedded samples were immunostained for melanocytic markers Melan-A, tyrosinase and HMB45, as well as for the B-cell/plasma cell markers and the immunoglobulin light chains using commercially available antibodies on a Dako or Ventana automated staining platform (see S2 Table for more details about the procedures). In addition, ISH for Epstein-Barr virus-encoded RNA (EBER1-2) and immunostaining for *Human Herpesvirus* 8 (HHV-8) was performed on the original biopsy material used for xenotransplantation (see S2 Table for more details about the procedures). Procedures involving human samples were approved by the UZ Leuven/KU Leuven Medical Ethical Committee (Commissie Medische Ethiek, approval number ML8713/S54185). All participants have given written informed consent before inclusion in the study. All signed informed consents have been collected and archived. Protocols for the generation, collection and recording of written informed consents have been formally approved by the supervising ethic committee/IRB.

### Animals and Husbandry

The 14 affected mice considered in this study were adult NOG females. All mice were part of the first transplant (F1) generation in the context of 6 different patient-derived metastatic melanoma experiments. S1 Table provides an overview of the entire patient-derived melanoma platform and highlights the distribution of the affected F1 animals in the context of the different PDTX experiments. In the F1 generation, material from metastatic melanoma biopsies is transplanted subcutaneously in the interscapular region of 6-week-old animals as described in the previous section. During the entire course of the experiment, mice were multiply housed (4 mice) in individual ventilated cages (Tecnilab, Blue Line IVCs) containing BK8/15 Lignocel wood bedding (J. Rettenmaier & Söhne GmbH) in a semi-SPF A2 facility at the Laboratory Animal Center KU Leuven. Food (standard rodent diet Ssniff R/M-H) and UV-filtered drinking water were provided *ad libitum*. Animal rooms were maintained at 22°C ± 2°C with a 45% and 70% relative humidity range, 50 air changes per hour and twelve-hour light/dark cycles. Mice were included in a health monitoring program developed in accordance with Federation of European Laboratory Animal Science Associations (FELASA) guidelines. Details regarding the pathogen status of the NOG colony are reported in S3 Table. Procedures involving animals were performed in accordance with the guidelines of the Catholic University of Leuven (KU Leuven) Animal Care and Use Ethical Committee which specifically approved this study (P147/2012).

### Mouse Necropsy, histopathology and immunohistochemistry

Mice were euthanized by CO2 asphyxiation followed by complete pathological examination including necropsy with dissection and histological analysis of the following organ and tissues: head *in toto*, skin (including site of transplantation and auricles), salivary glands, larynx and thyroids, trachea, lungs, heart, mediastinum, spleen, liver, pancreas, kidneys, esophagus and stomach, small intestine, large intestine, urogenital tract, cervical and tracheobronchial lymph nodes, sternum. Samples were immersion-fixed in 10% neutral buffered formalin (Sigma-Aldrich #HT501320), routinely processed for paraffin embedding, sectioned at 5 μm and stained with Hematoxylin (Diapath #C0302) and Eosin (Diapath #C0362). Serial sections obtained from representative samples were also stained by means of immunohistochemistry (IHC) or in situ hybridization (ISH) as detailed in the S2 Table. Head and sternum were decalcified in a 14% solution of Tetrasodium EDTA (VWR BDH Prolabo #20299291) for 15 days before processing and paraffin embedding.

### Mouse Hematology

Terminal blood collection was performed in 7 out of the 14 affected mice by means of cardiac puncture. Complete blood count (CBC) was then measured using scil Vet ABC hematology analyzer. Blood samples collected from 5 mice (i.e. 3 to 6 month-old non-transplanted and clinically healthy NOG females housed in the same colony) were also included in the analysis as controls.

### IGH and IGK clonality assay

IgH and IgK rearrangements were studied by multiplex PCR with BIOMED-2 primers using a BIOMED-2 PCR based protocol [22]. The final 50-μl reaction volume consists of 40-μl mastermix, 1-2U of TaqGold (Applied Biosystems) and 100 ng of DNA for both IgH and IgK reactions. The PCR reactions were performed on a ABI 9700 thermal cycler with the following amplification parameters; initial denaturation for 15 minutes at 94°C, followed by 32 cycli of 30 seconds at 95°C, 30 seconds at 60°C and 30 seconds at 72°C, with a final extension step of 10 minutes at 72°C. PCR product was added into the appropriate well of a 96-well PCR plate. A mix of HiDi Formamide and GeneScan-500 LIZ Size Standard was added into each well and mixed with 2 μl PCR product and finally loaded to the ABI3130xl analyzer. Paraffin sections from representative mouse lesions and matched frozen original tumor biopsies were used to isolate genomic DNA. The quality of the extracted DNA was excellent, showing an amplification up to 400 bp.

## Results

### Clinical history and necropsy findings in the affected NOG mice

**Fig 1:**
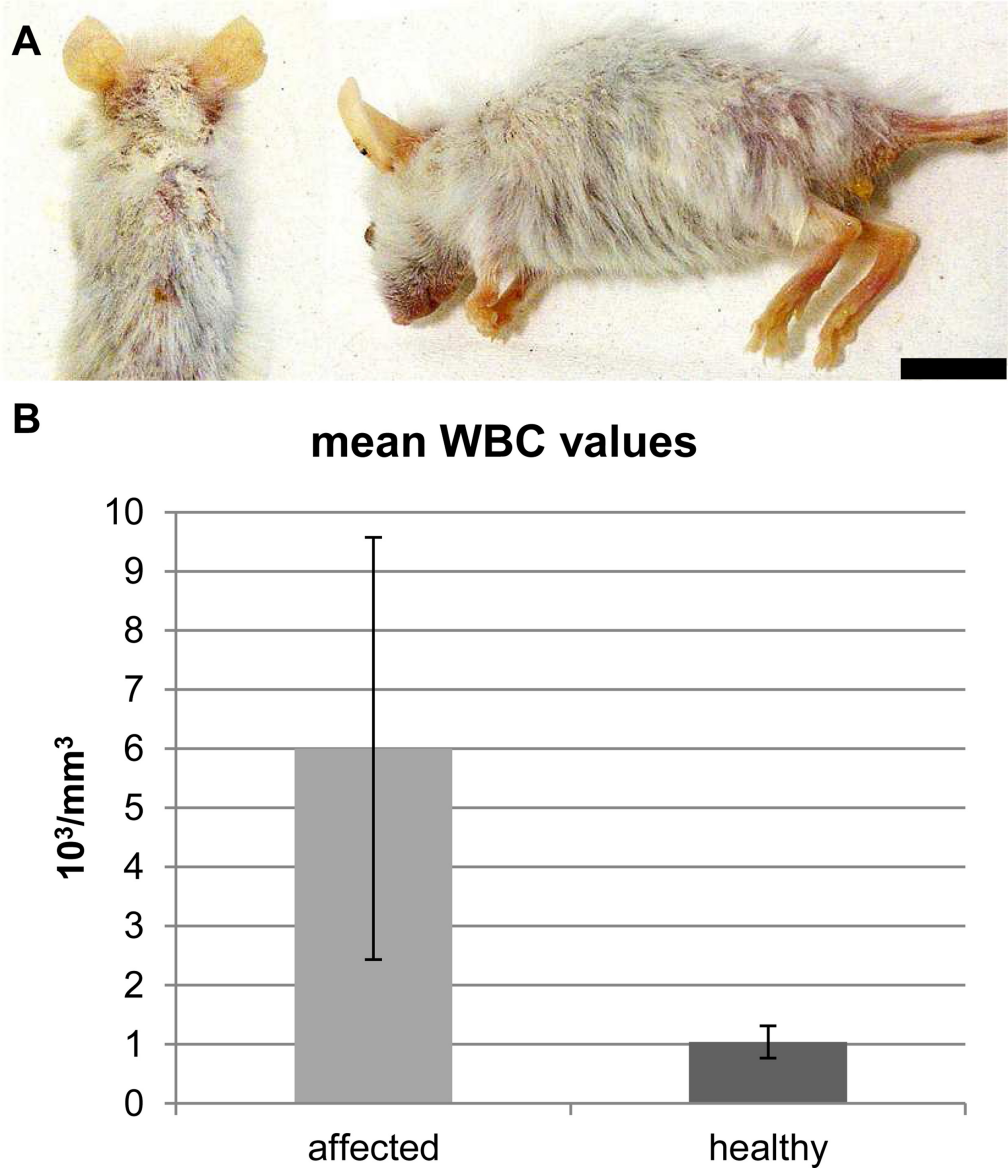
Clinical and necropsy findings in the affected NOG mice xenotransplanted with tissues from human metastatic melanoma. (A) 5-month-old female showing diffuse scaling and crusting dermatitis associated with alopecia. Note also the extreme emaciation of the animals and the complete absence of grossly detectable tumor growth in the interscapular region. Scale bar = 15 mm. (B) Total white blood cell count assessment in 7 affected NOG mice and 5 healthy matched controls from the same NOG colony. Affected NOG mice display a significant increase in white blood cell counts when compared to healthy controls (2-Tailed t-test, p = 0.012).

Fourteen out of 116 (12%) F1 NOG females coming from 6/30 (20%) transplantation experiments were submitted for complete necropsy and histopathology with a history of extensive scaling/crusting dermatitis and alopecia (13/14) associated with progressive wasting/emaciation (13/14) and poor/unsuccessful tumor engraftment (13/14) (Fig 1). Age of the affected F1 mice ranged between 2½ and 7 months. All the affected animals were transplanted with tissue from patient-derived metastatic melanoma at 6 weeks of age. Additional gross findings recorded during necropsy included small whitish subcutaneous/fascial nodules at site of transplantation (8/14), lymphadenomegaly mainly affecting cervical, tracheobronchial and renal lymph nodes (12/14), enlargement of the thymus (4/14), mild to moderate splenomegaly (11/14), mild hepatomegaly (6/14) and whitish mass in the cranial mediastinum (1/14) or renal hilum (1/14). It must be recalled that, because of their severe hypoplasia, thymus and lymph nodes in NOG mice are macroscopically inconspicuous. Hematological analysis performed on 7 of the affected animals also revealed a significant increase of white blood cell count when compared to matched-control mice (Fig 1). An overview of the distribution of the affected F1 animals in the context of the different PDTX experiments is given in S1 Table.

### Affected NOG mice suffer from a systemic immunoinflammatory disorder with features of xenogeneic graft versus host disease

**Fig 2:**
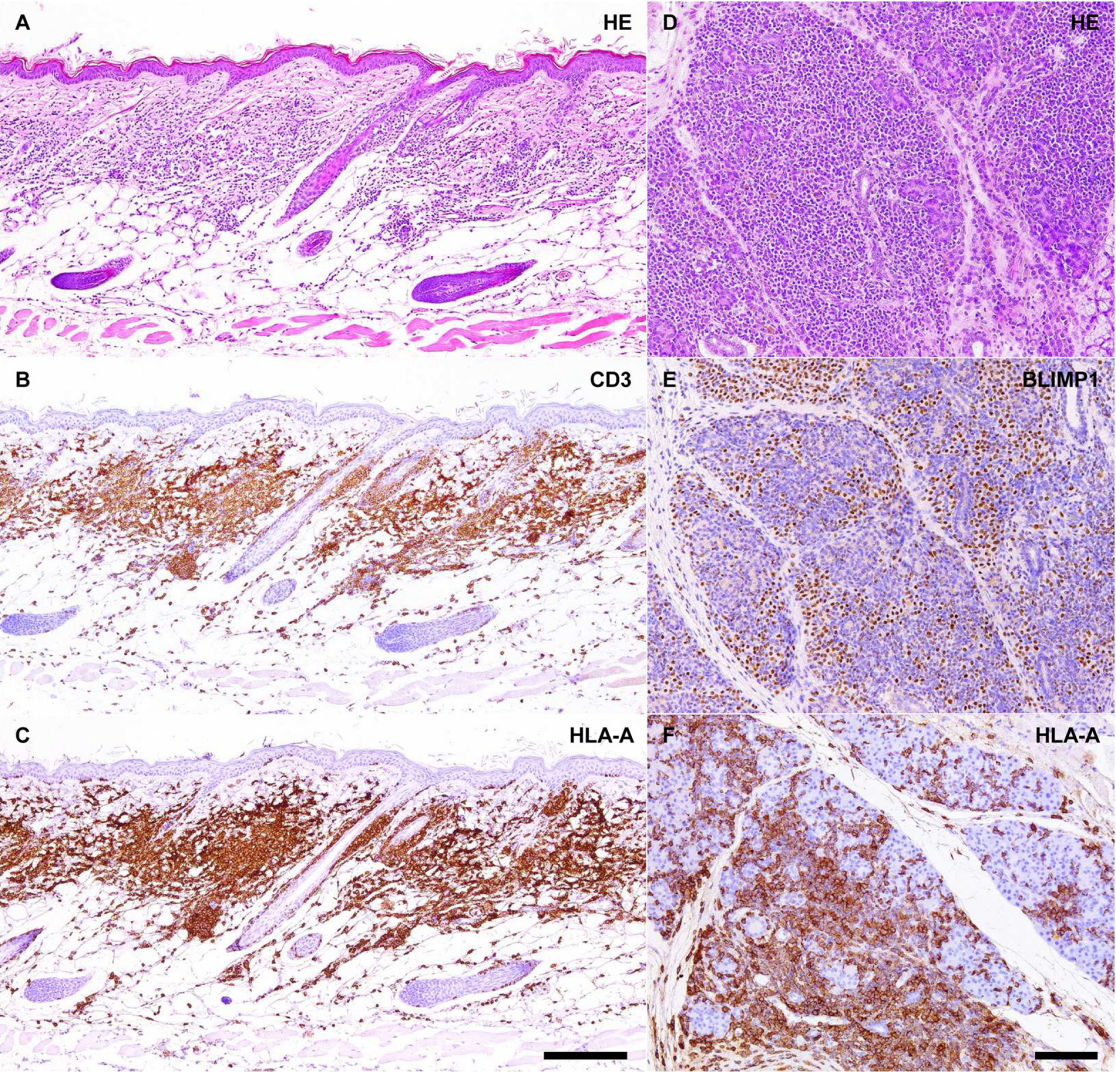
Affected NOG mice xenotransplanted with tissue from human metastatic melanoma suffer from chronic xenogeneic GVHD-like condition initiated by co-transplanted human immune cells populating the original tumor biopsy. (A-C) Chronic dermatitis with lichenoid interface pattern, virtually all the infiltrating immune cells co-express the T cell marker CD3 and the human-specific MHC class I molecule HLA-A. Hematoxylin an eosin (HE) staining and immunohistochemistry for CD3 and HLA-A, scale bar = 200 μm. (D-F) Dense plasma cell-rich infiltrates effacing the salivary gland parenchyma and showing prominent nuclear expression of the mature B cells/plasma cells marker BLIMP1, virtually all the infiltrating immune cells are also positive for the human-specific MHC class I molecule HLA-A. HE staining and immunohistochemistry for BLIMP1 and HLA-A, scale bar = 100 μm.

Microscopically, all the 14 affected animals exhibited infiltrates and/or proliferation of lymphoid cells in most of the analyzed organs and tissues with severe involvement of skin, salivary glands, thyroids and lungs and prominent expansion of lymph nodes, thymus and spleen (Fig 2 and S1 Fig). Most of the lesions were composed of a variable mixture of small to medium-sized lymphocytes and reactive plasma cells including numerous Mott cells. Skin lesions displayed a typical lichenoid interface pattern characterized by band-like infiltrates of lymphocytes and, to a lesser extent, plasma cells in the superficial-mid dermis with groups of small epitheliotropic lymphocytes multifocally invading the hyperplastic/hyperkeratotic epidermis and pilosebaceous units (Fig 2 and S1 Fig). Hydropic degeneration of the basal epidermal layer and lymphocytic satellitosis with basal/suprabasal keratinocyte apoptosis were also evident. A similar lesional pattern was also observed at the level of mucosal membranes lined by stratified squamous epithelium (e.g. oral cavity, esophagus, forestomach, vagina) (S1 Fig). Other organs/tissues showing different degrees of invasion and effacement of epithelial compartment by groups of small lymphocytes included salivary glands, olfactory mucosa, respiratory mucosa, thyroids, lungs (bronchioli), liver (bile ducts), pancreas (both exocrine and endocrine compartments) and proximal gastrointestinal tract (S1 Fig). Lymphoid infiltrates were often associated with variable degree of fibrosis. Most of the affected animals also displayed renal changes consistent with membranous/membranoproliferative glomerulonephritis (S1 Fig).

Two main hypotheses were formulated that could explain the pathogenesis of these reactive immunoinflammatory lesions: (i) development of immune cell “leakiness” in the mouse host with spontaneous generation of functional B and T cell clones; (ii) establishment of a chronic progressive form of human-into-mouse xenogeneic GVHD triggered by co-transplanted immune cells populating the patient-derived biopsy. To test these two hypotheses, tissue sections from representative lesions were immunostained with a monoclonal antibody that specifically recognizes human but not mouse major histocompatibility complex (MHC) class I. Representative lesions were also stained with a monoclonal antibody that specifically recognizes murine but not human CD45 Leukocyte Common Antigen (LCA). In addition, ISH for primate-specific Alu repetitive elements was performed to further test the human origin of the immune cells. The entire population of lymphoid cells in the infiltrates displayed strong membranous MHC class I immunoreactivity and diffuse positivity for Alu-ISH but invariably failed to express murine-specific CD45 LCA (Fig 2 and S2 Fig). These results unequivocally demonstrated the human origin of the infiltrates thus supporting the hypothesis of the development of a chronic GVHD-like condition initiated by human immune cells populating the original transplanted biopsy. In order to provide further evidence of the causative relationship between lymphoid infiltrates of human origin and clinical condition affecting xenotransplanted NOG mice, histological examination and IHC for the human-specific MHC class I were also conducted on selected tissue samples obtained from 9 clinically healthy NOG mice with successful metastatic melanoma engraftment. As expected, infiltrates and/or proliferation of human lymphoid cells were not detected in any of the examined organs and tissues including xenotransplanted tumor, skin, salivary glands, lungs, lymph nodes, spleen, liver, pancreas, kidneys and urogenital tract (S3 Fig).

To characterize the nature of the lymphoid infiltrates responsible for the development of the chronic GVHD-like condition, additional IHC stainings were performed. Not surprisingly, epitheliotropic infiltrates in the skin and other organs/tissues were almost entirely composed of CD3-positive T cells (Fig 2 and S4 Fig). On the contrary, plasma cell-rich infiltrates consisted of a variable mixture of CD20/PAX5/BLIMP1-positive mature B cells, BLIMP1/CD138/MUM1p-positive plasma cells and CD3-positive T cells (Fig 2). Co-localization studies using the human-specific MHC class I monoclonal antibody were also performed to further confirm the human origin of CD3-positive T cells and CD138-positive plasma cells populating the lymphoid infiltrates/proliferations (S4 Fig).

### Melanoma xenografts in affected NOG mice are effaced by human immune cells

**Fig 3:**
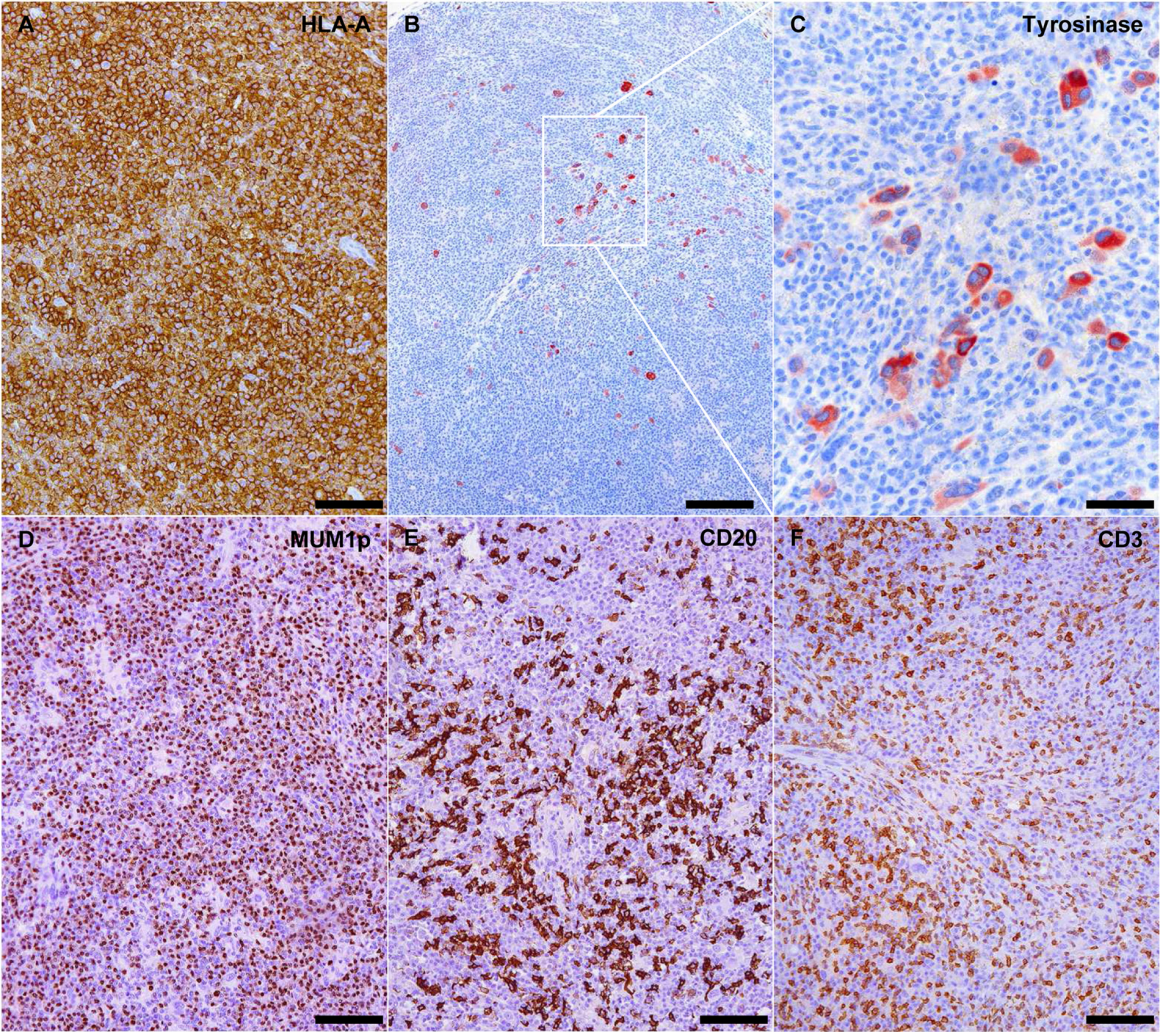
Metastatic melanoma xenografts in NOG mice affected by xenogeneic GVHD are effaced by plasma cell-rich lymphoid infiltrates. (A) Xenotransplanted metastatic melanoma is replaced by dense lymphoid infiltrates diffusely expressing human-specific MHC class I molecule HLA-A. HLA-A immunohistochemistry, scale bar = 100 μm. (B and C) Few residual groups of tyrosinase-positive melanoma cells are still detectable among the lymphoid infiltrates. Tyrosinase immunohistochemical staining, scale bar = 200 μm in B and 50 μm in C. Lymphoid infiltrates at the site of transplantation are primarily composed of MUM1p-positive plasma cells (D), CD20-positive mature B cells (E) and CD3-positive T cells. MUM1p, CD20 and CD3 immunohistochemistry, scale bar = 100 μm.

In 8 out of 14 mice affected by xenogeneic GVHD-like condition, microscopic changes at site of transplantation were characterized by dense plasma cell-rich lymphoid infiltrates of human origin with severe effacement of melanoma xenografts as demonstrated by the identification of scattered groups of Melan A, HMB45 and/or Tyrosinase-positive tumor cells interspersed among the reactive infiltrates (Fig 3). As observed also in other organs/tissues, plasma cell-rich infiltrates at site of transplantation were composed primarily of CD20/PAX5/BLIMP1-positive mature B cells, BLIMP1/CD138/MUM1p-positive plasma cells and CD3-positive T cells (Fig 3). Similar tumor-effacing plasma cell-rich lymphoid infiltrates were not identified in any of the original metastatic melanoma biopsies considered in this study.

### Development of monoclonal post-transplant lymphoproliferative disorders of donor origin in NOG mice suffering from xenogeneic GVHD-like condition

**Fig 4:**
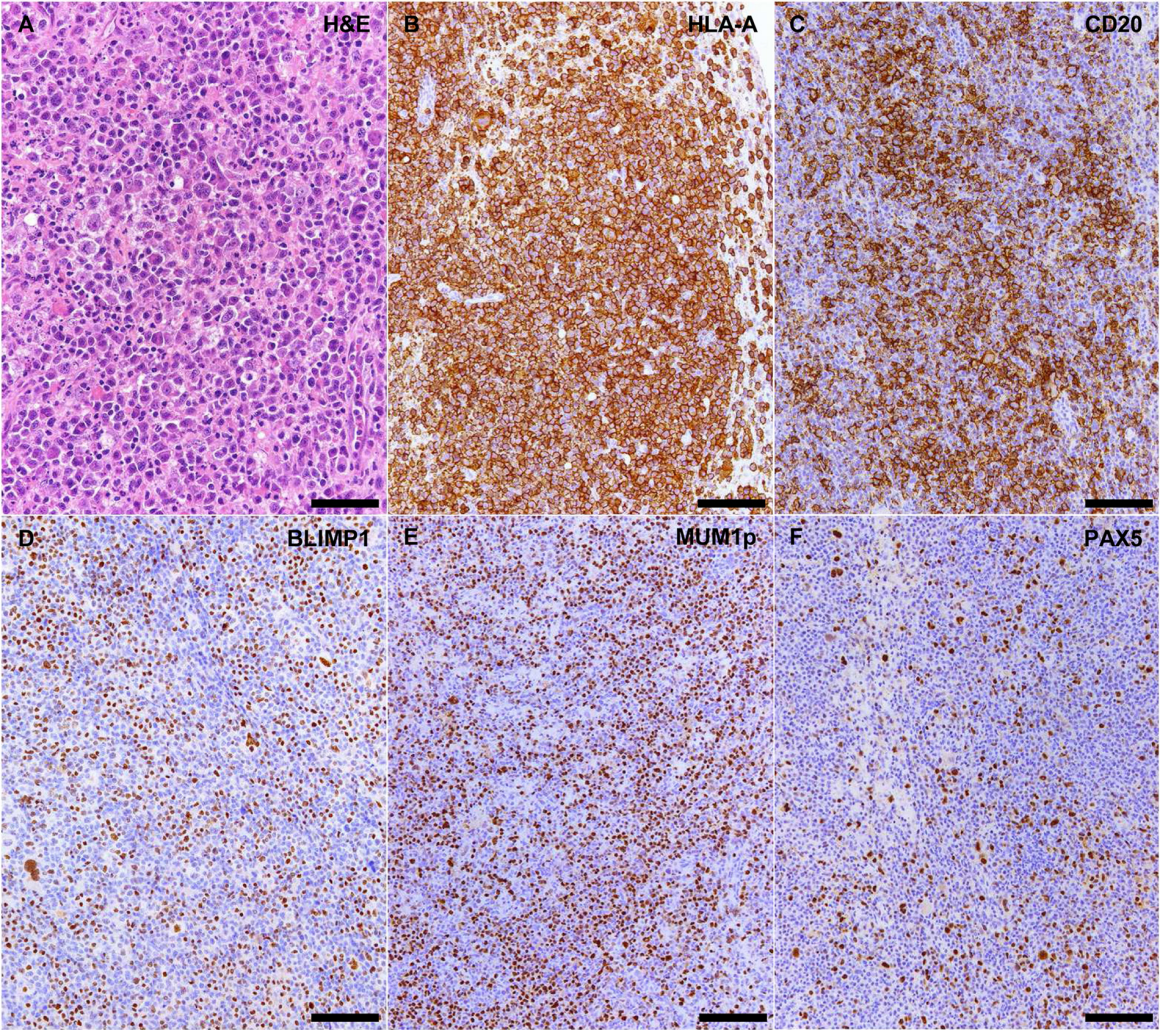
NOG mice suffering from xenogeneic GVHD-like condition occasionally develop atypical plasma cell-rich lymphoid infiltrates of donor origin. (A) Renal/perirenal plasma cell-rich infiltrates with findings of marked cytologic atypia. Hematoxylin and eosin staining (H&E), scale bar = 50 μm. (B) Human origin of the atypical lymphoid infiltrates is confirmed through the diffuse expression of human-specific MHC class I molecule HLA-A. HLA-A immunohistochemistry, scale bar = 100 μm. (C-F) Immunophenotypically the atypical lymphoid infiltrates are positive for markers of mature B cell/plasma cell differentiation including CD20, BLIMP1, MUM1p, PAX5. CD20, BLIMP1, MUM1p and PAX5 immunohistochemistry, scale bar = 100 μm.

**Fig 5:**
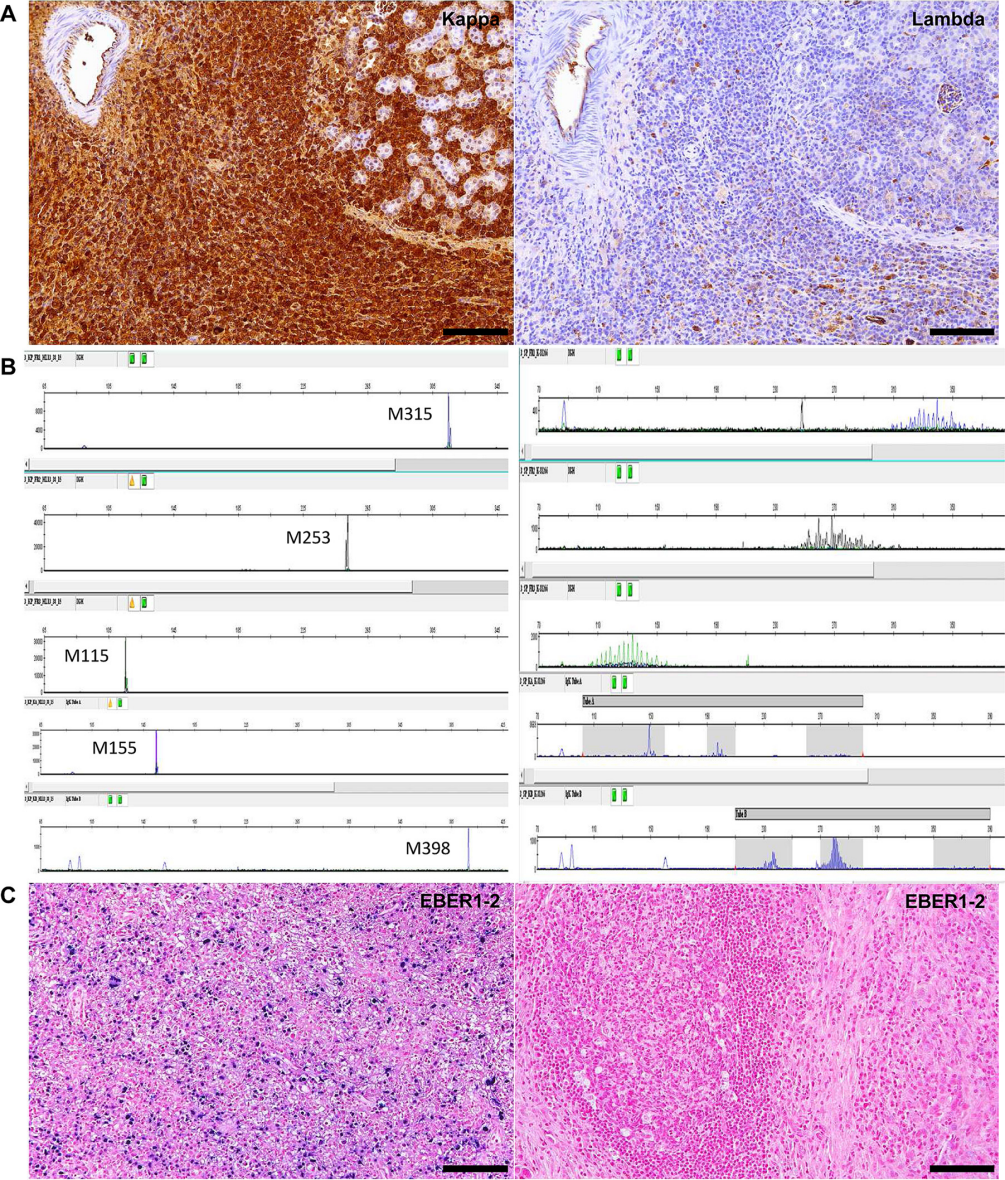
Atypical plasma cell-rich lymphoid infiltrates of donor origin in NOG mice xenotransplanted with human metastatic melanoma are monoclonal and reflect the development of post-transplant B cell/plasma cell malignancies associated with Epstein-Barr virus (EBV) reactivation. (A) The atypical plasma cell-rich infiltrates show kappa light chain restriction suggesting monoclonal B cell/plasma cell expansion. Kappa and Lambda light immunoglobulin chains immunohistochemistry, scale bar = 100 μm. (B) Atypical lymphoid infiltrates show a clear monoclonal pattern for all IgH and IgK frameworks confirming the development of post-transplant B cell/plasma cell neoplasms, fragment lenght for each monoclonal peak is indicated (left). On the contrary, matched original human tumor biopsy displays a polyclonal pattern which is consistent with the presence of reactive B cells/plasma cells (right). Multiplex PCR for IgH and IgK rearrangements. (C) Approximately 30–40% of the atypical B cells/plasma cells populating the monoclonal lymphoid proliferation display a strong EBV-encoded RNA (EBER1-2) hybridization signal (left). On the contrary no EBER1-2 signal is observed in the original tumor biopsy (right) suggesting a post-transplant reactivation of EBV. EBER1-2 in situ hybridization, scale bar = 100 μm.

In 2 out of the 14 animals affected by xenogeneic GVHD-like condition, we observed plasma cell-rich lymphoid infiltrates with findings of marked cytologic atypia (i.e. anisocytosis, anisokaryosis, cytomegaly, karyomegaly, multinucleation and increased numbers of mitoses with aberrant mitotic figures) expanding the mediastinum and renal hilum, respectively (Fig 4). Also in these cases, IHC for human-specific MHC class I and mouse-specific CD45 LCA confirmed the human origin of the atypical infiltrates. Lesions were also immunostained for Melan A, HMB45 and Tyrosinase but no positive cells were detected ruling out a possible combination between atypical metastatic melanoma cells accompanied by dense plasma cell-rich lymphoid infiltrates. On the contrary, atypical infiltrates were diffusely positive for markers of mature B cell/plasma cell differentiation (i.e. CD20, PAX5, BLIMP1, CD138 and MUM1p) (Fig 4) and showed kappa immunoglobulin light chain restriction suggesting a monoclonal expansion (Fig 5). In addition, plasma cells populating the atypical lesions showed an aberrantly high proliferative index as highlighted via duplex Ki67 and CD138 immunofluorescence (S5 Fig). The monoclonal nature of these atypical mature B cell/plasma cell proliferations/infiltrates was further demonstrated at molecular level (i.e. PCR for *IGH* and *IGL* loci rearrangement). Clonality profiles from mouse lesions and matched original tumors are presented in Fig 5. In both the atypical murine lymphoid infiltrates/proliferations, we observed a clear monoclonal pattern for *IGH* and *IGK*. Representative tissue samples with plasma cell-rich lymphoid infiltrates, considered reactive based on microscopic examination, were also selected (as controls) from NOG mice suffering from xenogeneic GVHD-like condition and confirmed to be polyclonal.

To rule out the possibility that lymphomatous cells were already coexisting with melanoma metastases, the two original tumor biopsies (i.e. melanoma metastases from an inguinal and axillary lymph node, respectively) were reviewed histopathologically, immunostained for immunoglobulin light chains and tested via PCR for *IGH* and *IGK* loci rearrangement. Microscopically, no features of cytologic atypia were observed in the residual nodal structures or in the scattered peritumoral infiltrates of lymphocytes and plasma cells. In both cases IHC failed to demonstrate immunoglobulin light chain restriction and PCR confirmed the polyclonal nature of B-cells and plasma cells populating the metastatic lesions.

Given the important role played by the Epstein-Barr Virus (EBV) and HHV-8 in the pathogenesis of post-transplant lymphomagenesis, original patient biopsies and corresponding lymphomatous lesions in the mouse were also assessed via ISH and IHC for EBER1-2 and HHV-8, respectively. All the samples tested negative for HHV-8 and original human metastases were also negative for EBER1-2. On the contrary, one of the affected mice revealed a strong EBER1-2 signal involving approximately 30–40% of the atypical B cells/plasma cells populating the monoclonal lymphomatous proliferation (Fig 5). Unfortunately there was no enough sample material left to confirm/rule out the implication of EBV in the B cell malignancy from the other affected mouse. Representative tissue samples with plasma cell-rich lymphoid infiltrates, considered reactive based on microscopic examination, were also selected (as controls) from NOG mice suffering from xenogeneic GVHD-like condition and confirmed to be negative for both the viruses.

Taken together, these results confirm the development of post-transplant malignancies of donor origin which are immunophenotypically consistent with diffuse large B cell lymphomas with plasma cell differentiation. In one case it was also possible to demonstrate the implication of EBV reactivation as the most likely cause of post-transplant B cell transformation.

## Discussion

A revolutionary aspect of NSG and NOG mice is the ability to promote efficient engraftment and expansion of diverse repertoires of leukocytes from different animal species including humans [11,23]. Thanks to this unique feature, both mouse lines are currently widely exploited in the preclinical field to generate mouse models with humanized immune system for studying immunopathological conditions [e.g. GVHD, delayed-type hypersensitivity (DTH)] [24,25], viral infections primarily targeting immune cells [e.g. human immunodeficiency virus (HIV) and EBV infections] [26]) and immunotherapeutic strategies (e.g. vaccine discovery and cancer immunotherapy) [27,28]. Although advantageous in diverse experimental settings, here we demonstrate that this capability of NOG mice to allow the engraftment of functional human immune cells may have unpredictable repercussion on the development of PDTX models. Co-transplanted lymphoid cells have indeed the potential to colonize systemically the recipient mouse, expand *in vivo* and eventually promote (i) immunoinflammatory disorders with features of xenogeneic GVHD, (ii) effacement of xenografted tumor at site of transplantation and (iii) monoclonal PTLD in the form of B cell lymphomas.

As it can be inferred from the complete overview of the different PDTX experiments included in our melanoma platform, clear correlations could not be found between the main clinicopathological characteristics of the original tumor (e.g. location of the metastasis, nature of the transplanted material in terms of tumor content and presence of lymphoid components, etc.) and the development of the post-transplant disorders described in the present study. The occurrence of the different post-transplant entities in mice receiving the same biopsy material was also unpredictable and not consistently reproducible. This is demonstrated by the fact that, within the same F1 transplant group, some animals only developed xenogeneic GVHD, some showed xenogeneic GVHD in combination with one or both of the other post-transplant disorders and some displayed a successful tumor engraftment without any clinical abnormality.

The development of xenogeneic GVHD has been already described as a major limitation in NSG mice receiving mononuclear cells from patients with acute myeloid leukemia (AML) without T cell depletion before transplantation [29]. In this experimental setting, NSG model proved to be unsuitable for preclinical applications as the high frequency of xenogeneic GVHD was consistently associated with failure of leukemic cell engraftment and premature mouse death. Similarly, we observed that more than 10% of NOG mice transplanted with biopsy material from metastatic melanoma exhibited unsuccessful tumor engraftment associated with diffuse dermatitis and progressive deterioration of health status ultimately requiring premature euthanasia. The comprehensive clinicopathological characterization of this entity revealed extensive immunoinflammatory changes in organs and tissues that are characteristically implicated during chronic GVHD (e.g. skin and adnexa, mucous membranes, salivary glands and portal spaces) [30]. In addition, the distinct patterns of lesion distribution (i.e. T cell infiltrates with lichenoid interface pattern in the skin and mucous membranes), coupled with the demonstration of the human origin of infiltrating lymphoid cells, provided unequivocal evidence for the development of human-into-mouse xenogeneic GVHD triggered by immune cells populating the transplanted tumor samples. Importantly, confirmation of the human origin of lymphoid cells definitely ruled out the possibility that these lesions may have resulted from spontaneous generation of mouse T and B cell (phenomenon also referred to as “leakiness”). Leakiness, which is a well-documented (usually age-related) limiting factor for tumor engraftment in immunocompromised mouse lines carrying the *Prkdc* ^*scid*^ mutation, is indeed reported as an exceedingly rare or nonexistent event in NOG or NSG mice [12,31,32].

It is currently unclear whether a correlation exists between the development of human-into-mouse xenogeneic GVHD and the nature of the transplanted tumor and/or tumor-associated lymphocyte repertoires. Melanoma is actually considered one of the most highly immunogenic tumors capable of inducing a strong immune response against a vast array of tumor-associated antigens [33]. In this context, it is interesting to note that xenogeneic GVHD also emerged as a major limiting factor in NSG mice bearing xenografts from established melanoma cell lines and receiving adoptive cell therapy with the melanoma-associated antigen MART-1-specific human T cells [28]. These observations, together with the findings described in our study, suggests that beside the classical xeno-immune response against foreign major and minor histocompatibility complexes [34], molecular mimicry between dominant melanoma-associated antigens and undetermined mouse epitopes may also play a role in the development of xenogeneic GVHD. Further supporting this view, significant expansion of co-transplanted lymphoid cells but lack of xenogeneic GVHD have been recently reported in NSG mice xenotransplanted with patient-derived non-small cell lung cancer which is notoriously regarded as poorly immunogenic tumor [15,33]. Another aspect that would definitely be very interesting to assess in a prospective study is whether the risk for xenogeneic GVHD is contributed by specific determinants present in the MHC haplotype of the donor [35].

In the clinical setting, melanoma invasion by plasma cells is an exceedingly rare finding that is occasionally described in primary cutaneous melanoma [36]. The same has been confirmed also in this study where none of the original metastatic melanoma biopsies exhibited this feature. Conversely, we observed that the majority of transplanted animals suffering from GVHD-like condition displayed unsuccessful melanoma engraftment associated with severe effacement of tumor xenografts by dense infiltrates of donor B-cells, plasma cells and T-cells. Although the exact pathogenesis of this finding remains largely unclear, possible speculations can be made to explain the extensive replacement of melanoma xenografts by co-transplanted immune cells. Considering the well-documented resistance of metastatic melanoma to antibody-mediated killing mechanisms [i.e. complement-dependent cytotoxicity (CDC), antibody-dependent cellular cytotoxicity (ADCC)] [37–39], development of an effective adaptive humoral immune attack against malignant cells after tumor transplantation into NOG recipients emerges as the most attractive scenario. This hypothesis actually opens interesting perspectives on whether, under the influence of a highly permissive recipient microenvironment [27], specific subsets of co-transplanted immune cells (which are normally repressed/quiescent in the original metastatic lesions) could have been selected to mount an effective humoral immunity circumventing melanoma immune escape strategies. In addition, xenotransplantation event might have played a role in the activation of a robust immune response against melanoma cells by unmasking novel dominant antigens that were concealed/segregated from the immune system in the original metastatic lesions. Although PDTXs in NSG and/or NOG mice are considered relatively stable models reflecting biologically the original tumor in the patient [4,5,8], profound variation in the spectrum of tumor-associated antigens represents indeed a well-described phenomenon which is frequently encountered in the context of xenotransplantation experiments [40–43]. Although in our study a humoral immune response appears to be the main mechanism responsible for the effacement of patient-derived melanoma xenografts, the heterogeneous population of T and B cells frequently observed at site of transplantation suggests that additional immune strategies may have also played a role. In this context, a recent investigation demonstrates that synergic interactions between 4-1BBL-expressing B cells and CD8-positive cytotoxic T cells can significantly contribute to reduce melanoma growth in syngeneic mouse models [44].

The development of a monoclonal PTLD of donor origin in 2 of the 14 NOG mice suffering from xenogeneic GVHD-like disease was another unanticipated finding. In both cases, immunohistological and molecular characterization of the atypical lymphoproliferative lesion confirmed the development of a mature B cell malignancy (most likely a DLBCL) with features of plasma cell differentiation [45]. EBV reactivation is responsible for B cell transformation in most of the reported clinical cases of post-transplant B cell lymphomas of donor origin [46–48]. Similarly, we were able to demonstrate reactivation of EBV infection as the most likely event driving post-transplant B cell lymphoma development in one of the two affected NOG mice. Strong EBER1-2 signal was indeed revealed in the atypical B cells/plasma cells populating the monoclonal lymphomatous proliferation whereas original tumor biopsy and reactive lymphoid infiltrates observed at site of transplantation or in the context of human-into-NOG mouse xenogeneic GVHD-like condition tested negative. Reactivation of EBV infection in co-transplanted human lymphocytes was also recently documented in a series of NOG or NSG-based PDTX experiments including breast, colorectal, gastric, lung and prostate [49,50]. In these latter works, EBV was identified by means of IHC and/or ISH in the context of lymphoproliferative lesions of B cell origin at the site of transplantation. Despite some aspects concerning the pathobiology of the lymphoproliferative lesions reported in these studies have not been fully elucidated (e.g. reactive versus neoplastic nature of the lymphoid proliferations, differentiation between co-transplanted human and native murine immune cells, evidence of lymphomatous proliferations in the original tumor biopsies and status of EBV infection in the human patients), similarities between our findings and their observations corroborate the general view that EBV may drive PTLD of donor origin in diverse NOG or NSG mouse-based PDTX experimental contexts [49–51].

In conclusion, the possibility for the development of xenogeneic GVHD driven by human immune cells populating the original tumor samples should be considered as a possible limitation in the context of NOG model-based PDTX experiments as this entity primarily impacts on the rate of tumor engraftment/growth and animal lifespan. The condition may also represent a major confounding factor for efficacy studies and personalized therapy trials where impaired tumor engraftment/growth can be potentially misinterpreted as positive response to treatment [7]. On the other hand, the replacement of the melanoma at the site of transplantation by a dense infiltrate of T-cells and plasma cells also marks an ongoing immune response against the tumor, resulting in a loss of melanoma cells, and thus may aid in the identification of novel tumor-antigens and establishment of effective immunotherapies. Lastly, the detailed characterization of B cell neoplasms of donor origin in our experimental setting reinforces the notion that NOG mice transplanted with patient-derived tumors may have a specific proclivity for the development of PTLD following the reactivation of EBV infection in the recipient host.

## Supporting Information

S1 Fig Histopathology of the immunoinflammatory lesions affecting NOG mice xenotransplanted with tissues from human metastatic melanoma. Dense infiltrates/proliferations of reactive lymphocytes and/or plasma cells with: (A) expansion of the fibrotic auricular dermis and epitheliotropic invasion of the hyperplastic/hyperkeratotic epidermis and pilosebaceous units, (B) expansion of cervical lymph node and invasion/effacement of the adjacent fibrotic parotid gland, (C) almost complete effacement of thyroid gland, (D) diffuse infiltration of pulmonary parenchyma, (E) multifocal infiltration of the renal cortex associated with membranous glomerulonephritis, (F) epitheliotropic invasion of the oral mucosa, (G) expansion of white pulp in the spleen [(H) note the undistinguishable hypoplastic white pulp in a non-affected NOG mice]. H&E staining. Scale bar = 100 μm (A, E, F), 200 μm (B, C) and 400 μm (D, G, H).
(TIF)

S2 Fig Demonstration via immunohistochemistry and *in situ* hybridization of the human origin of lymphoid infiltrates in the NOG mice affected by xenogeneic GVHD-like condition. (A and B) The great majority of immune cells in the perivascular pulmonary infiltrates are positive for primate-specific Alu repeats and human-specific MHC class I molecule HLA-A. (C) On the contrary, only scattered cells (most likely resident macrophages and dendritic cells) are labeled by the mouse specific CD45/LCA antibody. HLA-A and CD45/LCA immunohistochemistry and Alu repeats *in situ* hybridization, scale bar = 100 μm.
(TIF)

S3 Fig Demonstration via immunohistochemistry that lymphoid infiltrates of human origin are not present in tissues and organs obtained from clinically healthy NOG mice with successful metastatic melanoma engraftment. (A) Immune cells expressing the human-specific MHC class I molecule HLA-A are not evident in the salivary glands. (B) Metastatic melanoma xenograft with overlying skin, note that the xenotransplanted tumor diffusely expresses the human-specific MHC class I molecule HLA-A but no positive infiltrates of immune cells are detectable in the overlying skin or peritumoral soft tissues. HLA-A immunohistochemistry, scale bar = 200 μm.
(TIF)

S4 Fig Co-localization studies confirm the human origin of T lymphocytes and plasma cells in the lymphoid infiltrates affecting NOG mice xenotransplanted with tissues from human metastatic melanoma. Virtually all the infiltrating CD138-positive plasma cells and/or CD3-positive T cells also express the human-specific MHC class I molecule HLA-A. (A) Human plasma cells expanding the cervical lymph node of an affected NOG mouse. Duplex HLA-A and CD138 immunofluorescence, scale bar = 35 μm. (B) Epitheliotropic infiltrates of human T cells in the salivary gland of an affected NOG mouse. Duplex HLA-A and CD3 immunofluorescence, scale bar = 75 μm. (C) Prominent expansion of human T cells in the thymus of an affected NOG mouse. Duplex HLA-A and CD3 immunofluorescence, scale bar = 100 μm.
(TIF)

S5 Fig Atypical plasma cell-rich lymphoid infiltrates of donor origin in NOG mice xenotransplanted with metastatic human melanoma are characterized by exceptionally high proliferative activity. (A) CD138-positive plasma cells populating the atypical lymphoid infiltrates display an aberrantly high proliferative index. (B) Note the absence of Ki67-positive plasma cells in hepatic lesions characterized by non-atypical lymphoid infiltrates which were considered reactive based on microscopic examination. Duplex Ki67 and CD138 immunofluorescence, scale bar = 50 μm.
(TIF)

S1 Table Complete overview of the different PDTX experiments included in the melanoma platform. The table delineates distribution and frequency of the different of post-transplant disorders developed by xenotraspanted NOG mice how they correlate with the original tumor biopsies.
(XLSX)

S2 Table Details concerning reagents and procedures used for immunohistochemistry and in situ hybridization. (DOCX)

S3 Table Health report with the pathogen status of the NOG colony. (DOCX)
