# Supplementary figures and images for: Spontaneous Post-Transplant Disorders in NOD.Cg- Prkdcscid Il2rgtm1Sug/JicTac (NOG) Mice Engrafted with Patient-Derived Metastatic Melanomas
Source: PLoS One. 2015 May 21;10(5):e0124974. doi: 10.1371/journal.pone.0124974 (PMC4440639; doi:10.1371/journal.pone.0124974)

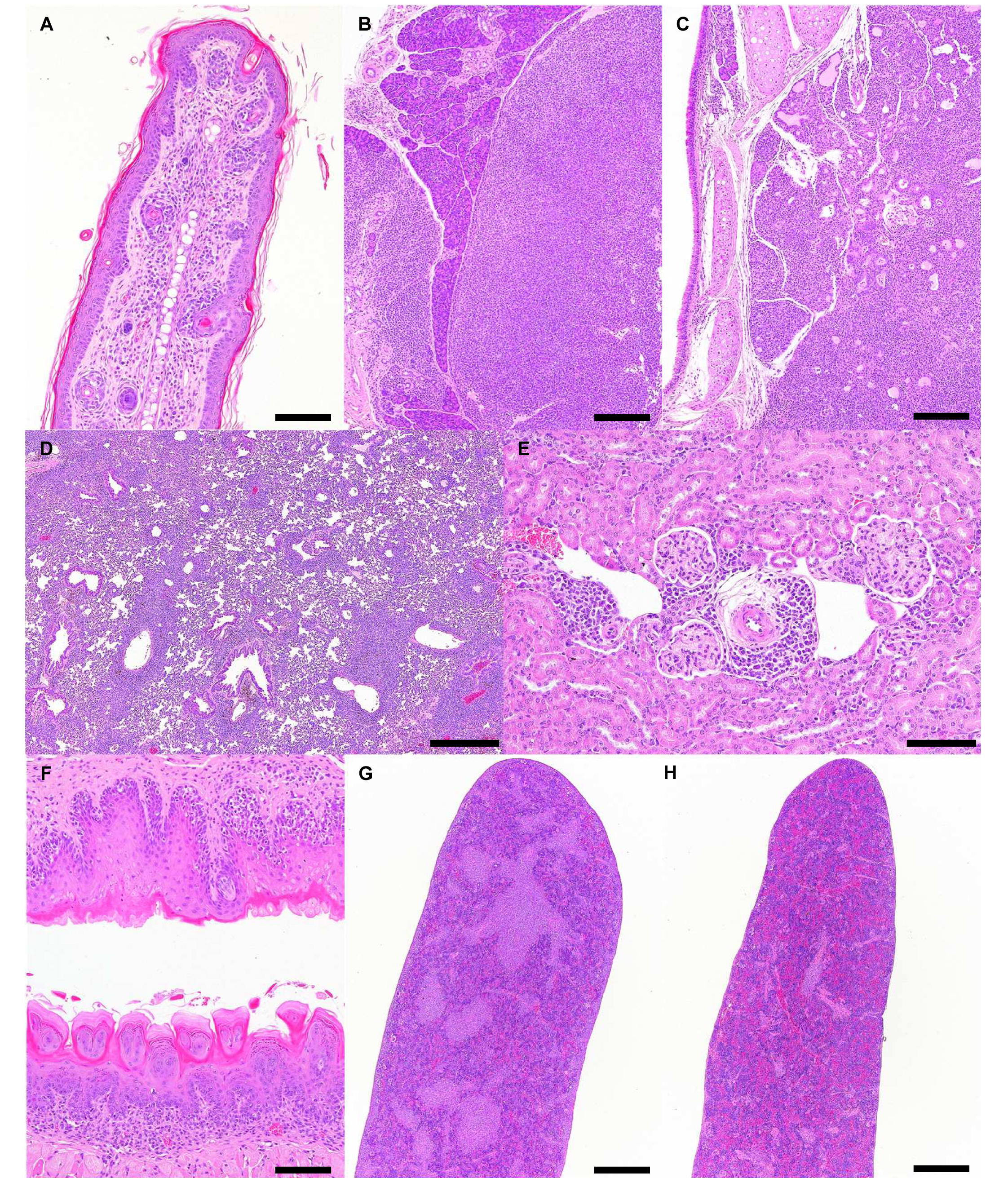

Supplement: S1 Fig — Dense infiltrates/proliferations of reactive lymphocytes and/or plasma cells with: (A) expansion of the fibrotic auricular dermis and epitheliotropic invasion of the hyperplastic/hyperkeratotic epidermis and pilosebaceous units, (B) expansion of cervical lymph node and invasion/effacement of the adjacent fibrotic parotid gland, (C) almost complete effacement of thyroid gland, (D) diffuse infiltration of pulmonary parenchyma, (E) multifocal infiltration of the renal cortex associated with membranous glomerulonephritis, (F) epitheliotropic invasion of the oral mucosa, (G) expansion of white pulp in the spleen [(H) note the undistinguishable hypoplastic white pulp in a non-affected NOG mice]. H&E staining. Scale bar = 100 μm (A, E, F), 200 μm (B, C) and 400 μm (D, G, H). (TIF) [file pone.0124974.s001.tif]

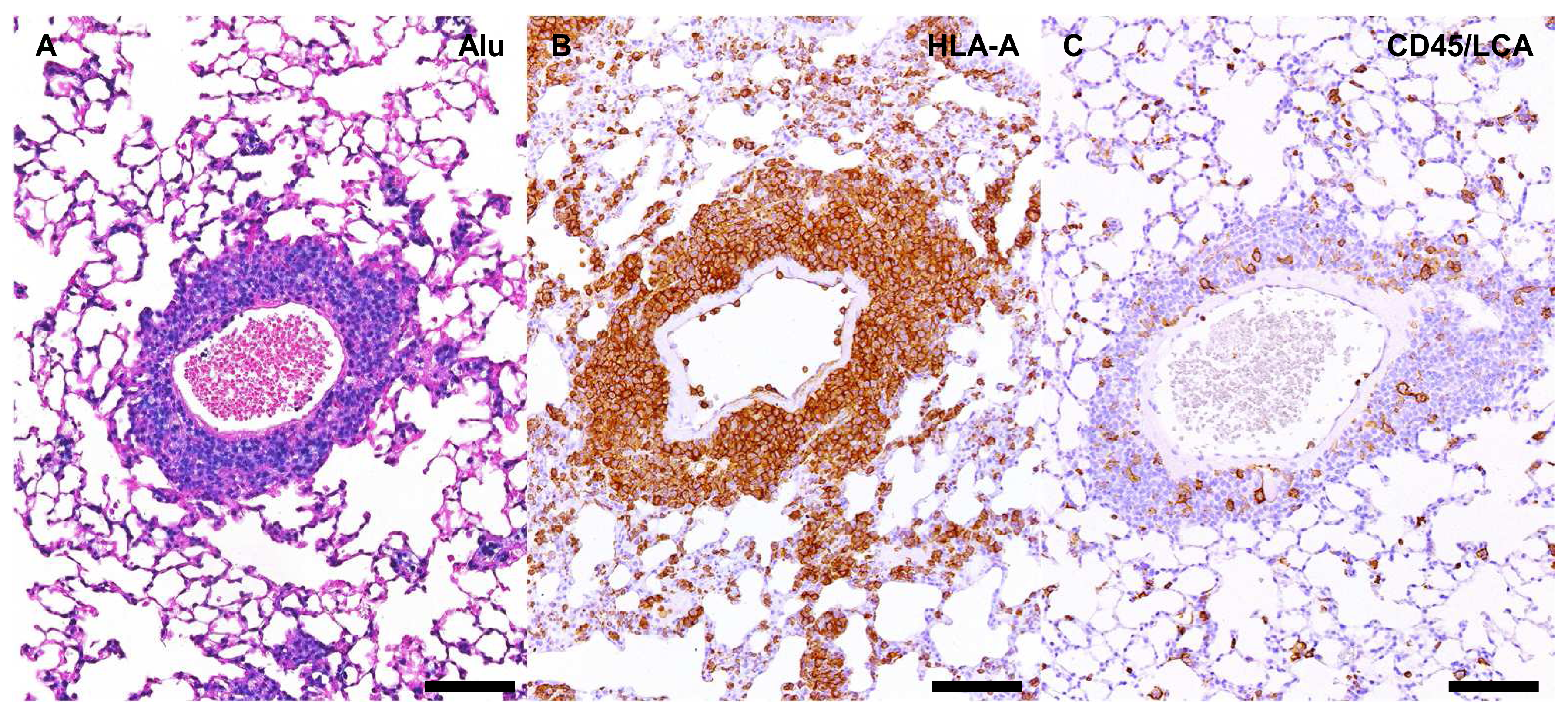

Supplement: S2 Fig — (A and B) The great majority of immune cells in the perivascular pulmonary infiltrates are positive for primate-specific Alu repeats and human-specific MHC class I molecule HLA-A. (C) On the contrary, only scattered cells (most likely resident macrophages and dendritic cells) are labeled by the mouse specific CD45/LCA antibody. HLA-A and CD45/LCA immunohistochemistry and Alu repeats in situ hybridization, scale bar = 100 μm. (TIF) [file pone.0124974.s002.tif]

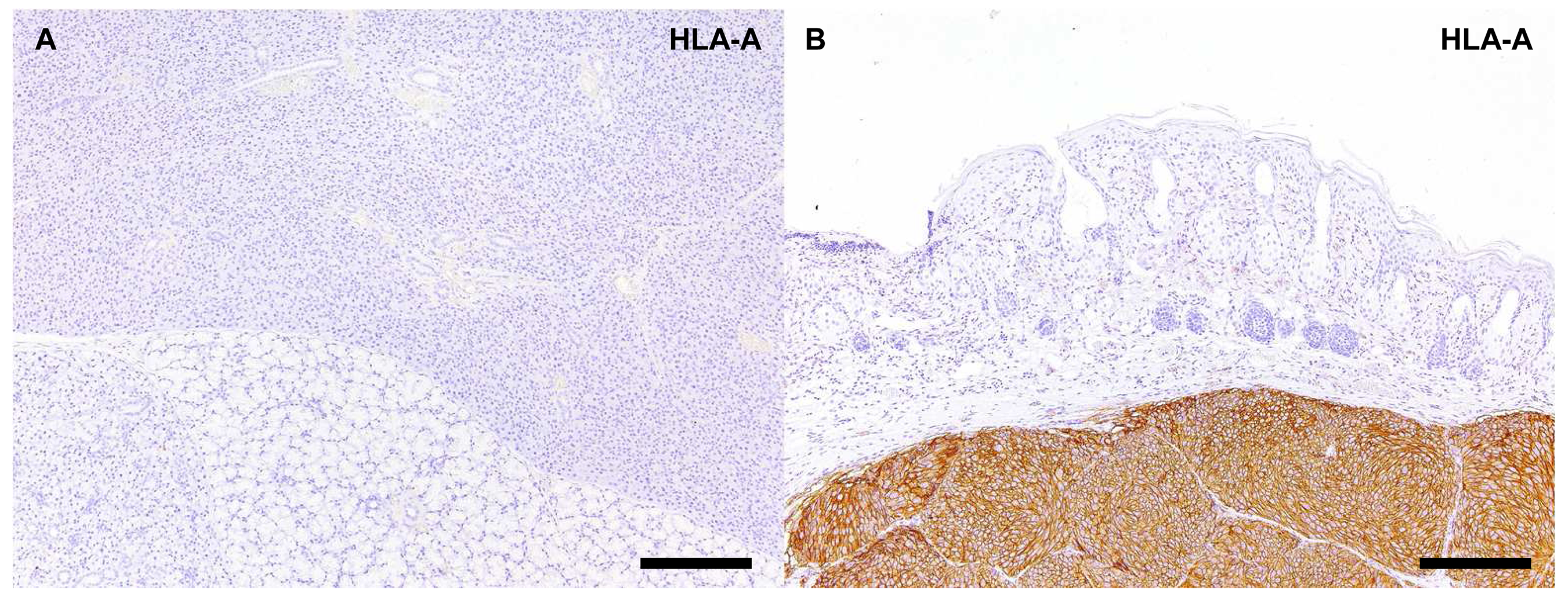

Supplement: S3 Fig — (A) Immune cells expressing the human-specific MHC class I molecule HLA-A are not evident in the salivary glands. (B) Metastatic melanoma xenograft with overlying skin, note that the xenotransplanted tumor diffusely expresses the human-specific MHC class I molecule HLA-A but no positive infiltrates of immune cells are detectable in the overlying skin or peritumoral soft tissues. HLA-A immunohistochemistry, scale bar = 200 μm. (TIF) [file pone.0124974.s003.tif]

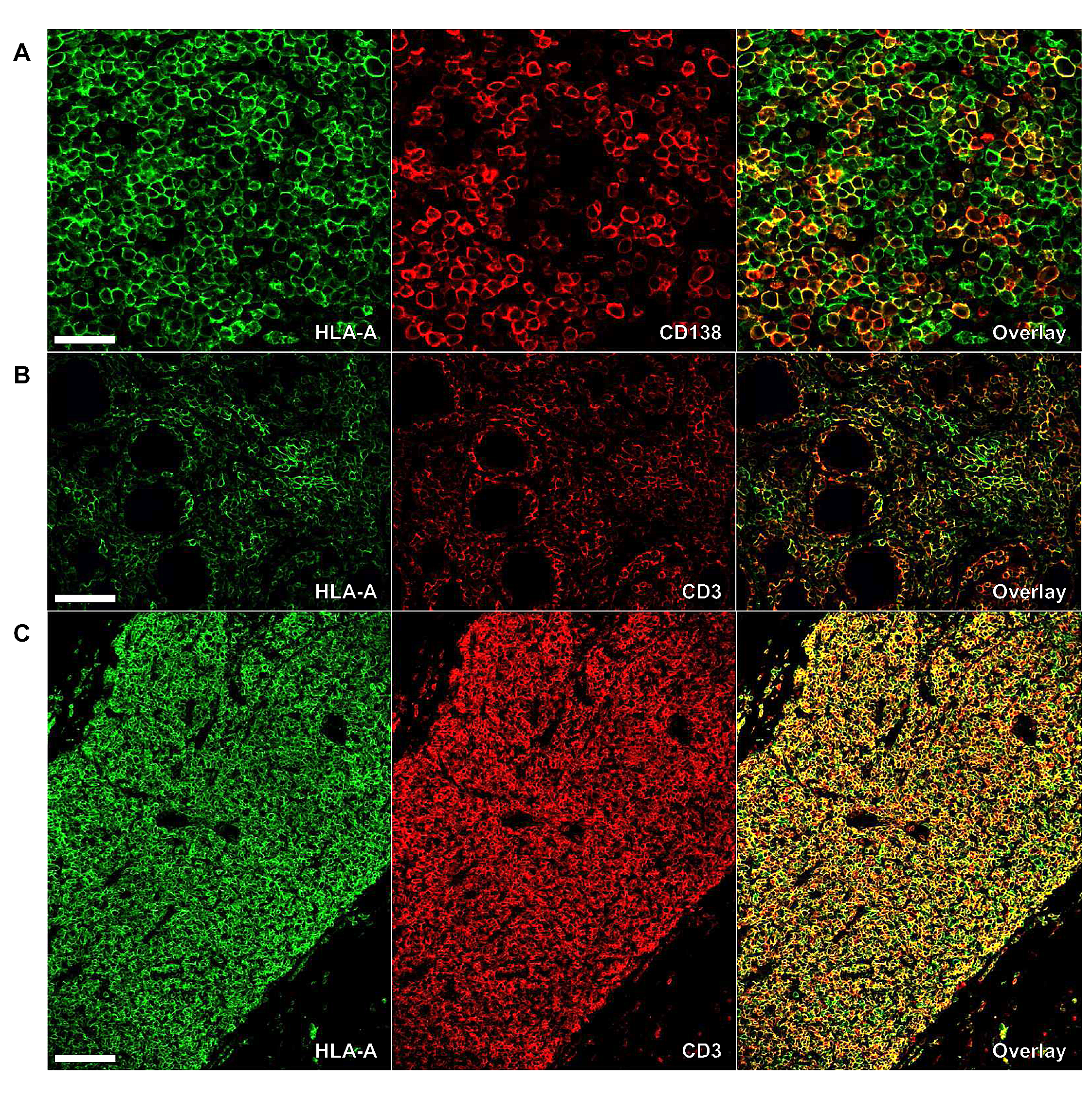

Supplement: S4 Fig — Virtually all the infiltrating CD138-positive plasma cells and/or CD3-positive T cells also express the human-specific MHC class I molecule HLA-A. (A) Human plasma cells expanding the cervical lymph node of an affected NOG mouse. Duplex HLA-A and CD138 immunofluorescence, scale bar = 35 μm. (B) Epitheliotropic infiltrates of human T cells in the salivary gland of an affected NOG mouse. Duplex HLA-A and CD3 immunofluorescence, scale bar = 75 μm. (C) Prominent expansion of human T cells in the thymus of an affected NOG mouse. Duplex HLA-A and CD3 immunofluorescence, scale bar = 100 μm. (TIF) [file pone.0124974.s004.tif]

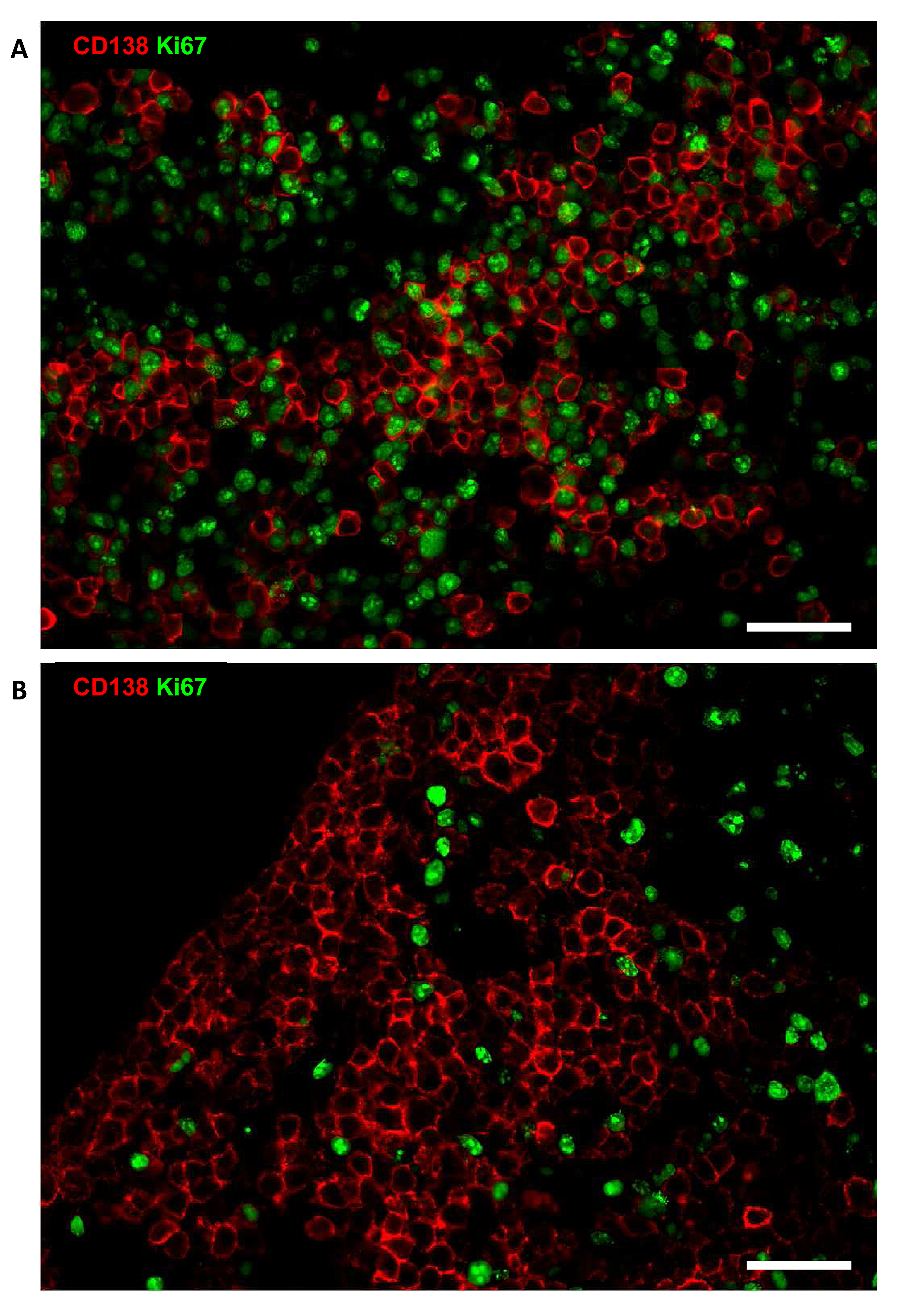

Supplement: S5 Fig — (A) CD138-positive plasma cells populating the atypical lymphoid infiltrates display an aberrantly high proliferative index. (B) Note the absence of Ki67-positive plasma cells in hepatic lesions characterized by non-atypical lymphoid infiltrates which were considered reactive based on microscopic examination. Duplex Ki67 and CD138 immunofluorescence, scale bar = 50 μm. (TIF) [file pone.0124974.s005.tif]
