# Supplementary material for: Spontaneous Post-Transplant Disorders in NOD.Cg- Prkdcscid Il2rgtm1Sug/JicTac (NOG) Mice Engrafted with Patient-Derived Metastatic Melanomas
Source: PLoS One. 2015 May 21;10(5):e0124974. doi: 10.1371/journal.pone.0124974 (PMC4440639; doi:10.1371/journal.pone.0124974)
Supplement: S2 Table — (DOCX) [file pone.0124974.s007.docx]

**S2 Table.** Details concerning reagents and procedures used for immunohistochemistry and *in situ* hybridization

| **Immunohistochemistry manual staining** | | | | |
| --- | --- | --- | --- | --- |
| **Target** | **Pretreatment** | **Primary antibody** | **Secondary antibody** | **Visualization system** |
| BLIMP1 | HIER pH6; 2100 Antigen Retriever | Rb mon (Cell Signaling Technology #9115); 1/1000; o/n incubation 4°C | EnVision™+/HRP (Dako K400311); 45’ RT | Immunoperoxidase, DAB chromogen reaction (Vector Labs ImmPACT™ DAB SK-4105) |
| CD3 | HIER pH6; 2100 Antigen Retriever | Rb poly (Dako A0452); 1/400; o/n incubation 4°C | EnVision™+/HRP (Dako K400311); 45’ RT | Immunoperoxidase, DAB chromogen reaction (Vector Labs ImmPACT™ DAB SK-4105) |
| CD3ɛ | HIER pH6; 2100 Antigen Retriever | Gt poly (Santa Cruz Biotechnology Sc-1127); 1/100; o/n incubation 4°C | Alexa Fluor® 555 Donkey Anti-Goat IgG H+L (Molecular Probes A-21432); 1/200; 45’ RT | Immunofluorescence |
| CD138 | HIER pH6; 2100 Antigen Retriever | Ms mon (Dako Clone MI15); RTU; o/n incubation 4°C | Alexa Fluor® 647 Donkey Anti-Mouse IgG H+L (Molecular Probes A-31571); 1/200; 45’ RT | Immunofluorescence |
| HLA-A | HIER pH6; 2100 Antigen Retriever | Rb mon (Abcam ab52922) 1/1200; o/n incubation 4°C | EnVision™+/HRP (Dako K400311); RTU; 45’ RT | Immunoperoxidase, DAB chromogen reaction (Vector Labs ImmPACT™ DAB SK-4105) |
| HLA-A | HIER pH6; 2100 Antigen Retriever | Rb mon (Abcam ab52922) 1/400; o/n incubation 4°C | Alexa Fluor® 488 Donkey Anti-Rabbit IgG H+L (Molecular Probes A-21206); 1/200; 45’ RT | Immunofluorescence |
| CD45 (LCA) | HIER pH6; 2100 Antigen Retriever | Rat mon (clone30-F11 - BD Pharmingen #553076) 1/1500; o/n incubation 4 °C | Biotinylated goat anti-Rat IgG (Vector Labs BA-9401); 1:100; 25’ RT | Immunoperoxidase, Avidin-Biotin Complex (Vector Labs, Vectastain Elite ABC kit PK-6100), DAB chromogen reaction (Vector Labs ImmPACT™ DAB SK-4105) |
| Ki67 | HIER pH6; 2100 Antigen Retriever | Rb mono (Thermo Scientific clone SP6 #RM-9106-S); 1/200; o/n incubation 4°C | Alexa Fluor® 488 Donkey Anti-Rabbit IgG H+L (Molecular Probes A-21206); 1/200; 45’ RT | Immunofluorescence |

| **Immunohistochemistry automated staining** | | | |
| --- | --- | --- | --- |
| **Target** | **Pretreatment** | **Primary antibody** | **Detection/visualization system** |
| Melan-A | Target Retrieval Solution, High pH (Dako K8004) | Ms mon (Dako Clone A103); RTU | Dako REAL™ Detection System APAAP, Mouse (K5000) |
| Tyrosinase | Target Retrieval Solution, High pH (Dako K8004) | Ms mon (Dako Clone T311); RTU | Dako REAL™ Detection System APAAP, Mouse (K5000) |
| Melanosome | Target Retrieval Solution, High pH (Dako K8004) | Melanosome (Dako Clone HMB-45); RTU | Dako REAL™ Detection System APAAP, Mouse (K5000) |
| CD138 | Target Retrieval Solution, High pH (Dako K8004) | Ms mon (Dako Clone MI15); RTU | Dako REAL™ Detection System APAAP, Mouse (K5000) |
| MUM1p | Target Retrieval Solution, High pH (Dako K8004) | Ms mon (Dako Clone MUM1p); RTU | Dako EnVision™ FLEX (K8010) |
| Pax-5 | Target Retrieval Solution, Low pH (Dako K8005) | Ms mon (Dako Clone DAK-Pax5); RTU | Dako EnVision™ FLEX (K8010) |
| CD20 | Target Retrieval Solution, High pH (Dako K8004) | Ms mon (Dako Clone L26); RTU | Dako EnVision™ FLEX (K8000) |
| Kappa light chains | Target Retrieval Solution, High pH (Dako K8004) | Rb poly (Dako Code IS506); RTU | Dako EnVision™ FLEX (K8010) |
| Lambda Light Chains | Target Retrieval Solution, High pH (Dako K8004) | Rb poly (Dako Code IS507); RTU | Dako EnVision™ FLEX (K8000) |
| HHV8/KSRV | Cell Conditioning Solution (Ventana Medical Systems #950-124) | Ms mon (Ventana Medical Systems Clone 13B10); RTU | UltraView Universal DAB Detection Kit (Ventana Medical Systems #760-500) |

| **In situ hybridization automated staining** | | | |
| --- | --- | --- | --- |
| **Target** | **Pretreatment** | **Probe** | **Detection/visualization system** |
| EBV (EBER 1 and 2) | Cell Conditioning Solution (Ventana Medical Systems #950-124) | INFORM EBER Probe (Ventana Medical Systems #950-124) | ISH iVIEW Blue Detection Kit (Ventana Medical Systems #800-092) |
| Alu repeats | Cell Conditioning Solution (Ventana Medical Systems #950-124) | Alu Positive Control Probe (Ventana Medical Systems #800-2845) | ISH iVIEW Blue Detection Kit (Ventana Medical Systems #800-092) |
