# Supplementary material for: Spontaneous Post-Transplant Disorders in NOD.Cg- Prkdcscid Il2rgtm1Sug/JicTac (NOG) Mice Engrafted with Patient-Derived Metastatic Melanomas
Source: PLoS One. 2015 May 21;10(5):e0124974. doi: 10.1371/journal.pone.0124974 (PMC4440639; doi:10.1371/journal.pone.0124974)
Supplement: S3 Table — (DOCX) [file pone.0124974.s008.docx]

**S1 Table:** Health report with the pathogen status of the NOG colony

| **Pathogen** | **Test Frequency** | **Test Method** | **Result** |
| --- | --- | --- | --- |
| **Virus** |  |  |  |
| Minute Virus of Mice (MVM) | Monthly | ELISA | negative |
| Mouse Encephalomyelitis Virus (GDVII)/ Rat Theilovirus (RTV) | Monthly | ELISA | negative |
| Mouse Hepatitis Virus (MHV) | Monthly | ELISA | negative |
| Mouse Parvovirus (MPV) | Monthly | ELISA | negative |
| Mouse Rotavirus (EDIM) | Monthly | ELISA | negative |
| Murine Norovirus (MNV) | Monthly | ELISA | negative |
| Pneumonia Virus of Mice (PVM) | Monthly | ELISA | negative |
| Ectromelia Virus | Quarterly | ELISA | negative |
| Hantaan Virus | Quarterly | ELISA | negative |
| K Virus | Quarterly | ELISA | negative |
| Lymphocytic Choriomeningitis Virus (LCM) | Quarterly | ELISA | negative |
| Mouse Adenovirus I and II (FL and K87) | Quarterly | ELISA | negative |
| Mouse Cytomegalovirus (MCMV) | Quarterly | ELISA | negative |
| Polyoma Virus | Quarterly | ELISA | negative |
| Reovirus (Reo 3) | Quarterly | ELISA | negative |
| Sendai Virus | Quarterly | ELISA | negative |
| Thymic Virus | Semi | IFA | negative |
| Lactate Dehydrogenase Elevating Virus (LDHV) | Annual | CHEM | negative |
| **Bacteria, Mycoplasma, Fungi** |  |  |  |
| Beta hemolytic Streptococcus (non Group D) | Quarterly | CULT | negative |
| Bordetella bronchiseptica | Quarterly | CULT | negative |
| Cilia Associated Respiratory Bacillus (CARB) | Quarterly | ELISA | negative |
| Citrobacter rodentium | Quarterly | CULT | negative |
| Clostridium piliforme | Quarterly | ELISA | negative |
| Corynebacterium bovis | Quarterly | CULT | negative |
| Corynebacterium kutscheri | Quarterly | CULT | negative |
| Helicobacter spp. | Quarterly | PCR | negative |
| Klebsiella oxytoca | Quarterly | CULT | negative |
| Klebsiella pneumoniae | Quarterly | CULT | negative |
| Mycoplasma pulmonis | Quarterly | ELISA | negative |
| Pasteurella multocida | Quarterly | CULT | negative |
| Pasteurella pneumotropica | Quarterly | CULT | negative |
| Pneumocystis spp. | Quarterly | PCR | negative |
| Proteus spp. | Quarterly | CULT | negative |
| Pseudomonas aeruginosa | Quarterly | CULT | negative |
| Salmonella spp. | Quarterly | CULT | negative |
| Staphylococcus aureus | Quarterly | CULT | negative |
| Streptococcus pneumoniae | Quarterly | CULT | negative |
| Segmented Filamentous Bacteria | Annual | PCR | negative |
| Streptobacillus moniliformis | Annual | PCR | negative |
| **Parasites** |  |  |  |
| Aspiculuris tetraptera | Quarterly | MICR | negative |
| Eimeria spp. | Quarterly | FLOT | negative |
| Encephalitozoon cuniculi | Quarterly | ELISA | negative |
| Entamoeba muris | Quarterly | MICR | negative |
| Giardia muris | Quarterly | MICR | negative |
| Hymenolepis spp. | Quarterly | FLOT | negative |
| Myobia musculi | Quarterly | MICR | negative |
| Myocoptes musculinus | Quarterly | MICR | negative |
| Ornithonyssus bacoti | Quarterly | MICR | negative |
| Polyplax spinulosa | Quarterly | MICR | negative |
| Psorergates simplex | Quarterly | MICR | negative |
| Radfordia affinis | Quarterly | MICR | negative |
| Rodentolepis spp. | Quarterly | FLOT | negative |
| Spironucleus muris | Quarterly | MICR | negative |
| Syphacia spp. | Quarterly | MICR-PCR | negative |
| Trichomonads | Quarterly | MICR | negative |
| **Restricted Flora (RF)** |  |  |  |
| Beta hemolytic Streptococcus (non Group D) - RF | Monthly | CULT | negative |
| Klebsiella oxytoca - RF | Monthly | CULT | negative |
| Klebsiella pneumoniae - RF | Monthly | CULT | negative |
| Pseudomonas aeruginosa - RF | Monthly | CULT | negative |
| Staphylococcus aureus -RF | Monthly | CULT | negative |

IFA= Immunofluorescent Assay; Cult= Culture; MICR= Microscopy; FLOT= Flotation
